# Transcriptional Landscape of Cotton Fiber Development and Its Alliance With Fiber-Associated Traits

**DOI:** 10.3389/fpls.2022.811655

**Published:** 2022-02-24

**Authors:** Priti Prasad, Uzma Khatoon, Rishi Kumar Verma, Shahre Aalam, Ajay Kumar, Debashish Mohapatra, Parthasarthi Bhattacharya, Sumit K. Bag, Samir V. Sawant

**Affiliations:** ^1^Division of Molecular Biology and Biotechnology, CSIR-National Botanical Research Institute, Lucknow, India; ^2^Academy of Scientific and Innovative Research (AcSIR), Ghaziabad, India; ^3^Department of Botany, University of Lucknow, Lucknow, India; ^4^Tierra Seed Science Pvt. Ltd., Hyderabad, India

**Keywords:** data-mining, stage-specific modules, cell commitment, *cis*-regulatory variation, fiber development, nCounter

## Abstract

Cotton fiber development is still an intriguing question to understand fiber commitment and development. At different fiber developmental stages, many genes change their expression pattern and have a pivotal role in fiber quality and yield. Recently, numerous studies have been conducted for transcriptional regulation of fiber, and raw data were deposited to the public repository for comprehensive integrative analysis. Here, we remapped > 380 cotton RNAseq data with uniform mapping strategies that span ∼400 fold coverage to the genome. We identified stage-specific features related to fiber cell commitment, initiation, elongation, and Secondary Cell Wall (SCW) synthesis and their putative cis-regulatory elements for the specific regulation in fiber development. We also mined Exclusively Expressed Transcripts (EETs) that were positively selected during cotton fiber evolution and domestication. Furthermore, the expression of EETs was validated in 100 cotton genotypes through the nCounter assay and correlated with different fiber-related traits. Thus, our data mining study reveals several important features related to cotton fiber development and improvement, which were consolidated in the “CottonExpress-omics” database.

## Introduction

A transcriptome is a repertoire for protein-coding and non-coding RNAs of different tissues that are crucial in delineating the molecular basis of any phenotypic plasticity. Since the last decade, more than 175 thousand RNAseq data of several plant species have been deposited in Sequence Read Archive (SRA) alone. Thus, making sense of such public archives for reproducibility and reusability of data was highly recommended for providing new biological insights (Rung and Brazma, 2013). Recently, refining of *Arabidopsis* genome has been achieved through the reuse of tissue-specific online submitted RNAseq libraries, which yielded 635 novel genes and 452 obsolete protein-coding genes (Cheng et al., 2017). Similarly, the co-expression network analysis of publicly available transcriptome data in *Arabidopsis* resulted in identifying trait-related modules (Liu et al., 2019) and the nitrate transporter in different tissues (He et al., 2016), whereas the meiosis-related modules were identified in hexaploid wheat (Alabdullah et al., 2019). The reanalysis of RNAseq datasets leads to the identification of “pan-network” and “core-network” for a condition-specific gene expression in Arabidopsis (He and Maslov, 2016; Mercatelli et al., 2020). In another study, pathogenesis-related RNAseq data were curated to provide cues for plant immunity responses in *Arabidopsis*, maize, wheat, and rice (Qi et al., 2018).

Cotton is one of the most important natural fiber crops, cultivated in more than 70 countries around the world, which contributed to 95% of global cotton production (Chen et al., 2007). The genus *Gossypium* is composed of 45 diploid and seven tetraploid species (Yuan et al., 2021). Tetraploid cotton species are natural allotetraploids formed by interspecific hybridization of A and D diploid genomes followed by polyploidization. The significant evolutionary pressure involving genetic and epigenetic modifications in the cotton genome is thought to be responsible for the domestications of modern-day cotton having much elongated and strengthened fiber (Paterson et al., 2012; Guan et al., 2014; Song et al., 2017; Li et al., 2019; Huang et al., 2021). Biologically, fibers are single-cell seed trichome that originates at the outer integument of the ovule surface in a highly polarized manner. Thus, they provide a model system for studying cell commitment, elongation, and cell wall deposition (Kim and Triplett, 2001). Fiber development involves four distinguished but overlapping stages, *viz.*, initiation, elongation, secondary cell wall biosynthesis, and maturation. The fate of ovule epidermal cells is committed before and/or on the day of anthesis, which is coordinated by different regulation machinery (Qin and Zhu, 2011; Zhang et al., 2017; Prakash et al., 2020; Tian and Zhang, 2021). Thus, we named this stage as a fiber commitment stage, which ultimately decided the total fiber yield (Wang L. et al., 2021). Cotton fiber yield and its quality are essential agronomical traits, and thereby, significant efforts have been made to understand its biological mechanisms. The two independent groups sequenced the draft genome of *Gossypium hirsutum* genome in 2015 (Li et al., 2015; Zhang et al., 2015) that revolutionized the cotton research, and resequencing efforts have also significantly improvised the reference genome (Hu et al., 2019; Wang et al., 2019; Yang et al., 2019; Chen et al., 2020; Huang et al., 2020). The high-quality assembly provides reliable genomic information like genome architecture, gene annotation, gene expansion during the cotton speciation and domestication processes.

However, before the cotton genome sequencing projects, numerous studies unravel the molecular mechanisms of cotton fiber development (Arpat et al., 2004; Wilkins and Arpat, 2005; Samuel Yang et al., 2006; Shi et al., 2006; Udall et al., 2006, 2007; Taliercio and Boykin, 2007; Liu et al., 2012; Gilbert et al., 2013; Salih et al., 2016). The significant RNAseq efforts were also examined to understand the fiber-specific expression dynamics in contrasting cotton genotypes (Nigam et al., 2014; Qin et al., 2019), fuzzless/lintless mutant (*fl*) (Wang et al., 2010; Ma Q. F. et al., 2016), Ligon lintless (Li1), and other mutant lines (Ding et al., 2014; Salih et al., 2016). Additionally, transcript profiling was used to quantify the gene expression changes at different developmental stages (Naoumkina et al., 2014; Wang et al., 2015; Guo et al., 2016; Hu et al., 2018; Ando et al., 2021; Li et al., 2021), which was further used for gene model prediction (Keilwagen et al., 2018). Thus RNAseq study can be broadly categorized before the availability of the draft cotton genome (Peng et al., 2014; Zhang et al., 2016; Parekh et al., 2018) and post-sequencing (Ma D. et al., 2016; Fang et al., 2017; Ma et al., 2018; Qin et al., 2019). The methodological variation in RNA sample preparation, sequencing technology, diverse mapping algorithm, and gene expression quantification leads to creating an obscure picture for defining the fiber dynamics at different fiber developmental stages (Corchete et al., 2020). Nevertheless, no coordinated efforts took into account to make use of publicly available RNAseq data to mine the fiber-specific features in a homogenous computational pipeline (Corchete et al., 2020).

In our current study, we used the high-quality reference genome of *Gossypium hirsutum* (Wang et al., 2019) and strengthened our understanding at the transcript level by reusing the publicly available cotton transcriptome data. We processed and clustered RNAseq data of different tissues by making use of a high-performance computing (HPC) facility. The clustering based on the coexpression network analysis identified fiber-specific genes that might have a distinct role at different stages of fiber development. The subgenome biased expression was also observed in our data with significant motif variation. Our comprehensive computational effort leads to identifying the stage-specific modules and Exclusively Expressed Transcripts (EETs). The expression level of identified EETs in 100 cotton genotypes and their correlation with different fiber quality and yield-related traits substantiates the importance of RNAseq data mining in cotton fiber development.

## Materials and Methods

### Differential Quantification and Cluster Analysis of Cotton Transcriptome Data

More than 380 mock-treated or untreated cotton RNAseq datasets of 40 SRA accessions were collected from the NCBI (Supplementary File 1). These datasets, having 175 bp average read length, comprised 18 tissues and 34 cultivars. SRA data were quality checked followed by the adaptor trimming by the Trimmomatic tool (Bolger et al., 2014) wherever required. Eight billion filtered reads were mapped on the *Gossypium hirsutum* reference genome (Wang et al., 2019) through the STAR aligner (Dobin et al., 2013) with default settings. Three hundred thirty-six samples of selected five tissues (ovule, fiber, leaf, root, and seed) were grouped into two major tissues: OF (ovule + fiber) and LRS (leaf + root + seed) for fiber-specific differential quantification using the Ballgown R package (Pertea et al., 2016). Differentially expressed genes (DEGs) were further subjected to the Weighted Gene Co-expression Network Analysis (Langfelder and Horvath, 2008) that was categorized into different modules. Block-wise modules were used with the soft threshold power of 10 to generate the module-wise clustering where the minimum module size was set to 10 with unsigned Topological Overlap Matrix. Clustering was based on co-expression behavior among the genes. By defining the degree of centrality between genes, the hub gene was also identified for these modules, which were eventually visualized through the Cytoscape plugin (Paul Shannon et al., 1971).

### Consolidation of Different DPAs Into Four Overlapping Stages

To check the expression profiling of different modules in fiber development stages, we consolidated the uniquely mapped reads from publicly available SRA datasets (Supplementary File 2) in four major subsets according to the different DPAs, viz: “−3 DPA,” “−1 DPA,” “0 DPA” into commitment, “1 DPA,” “3 DPA” were merged into initiation stages, “5 DPA,” “8 DPA,” “10 DPA,” “12 DPA,” “14 DPA,” “15-16DPA,” “18 DPA,” “20 DPA” grouped for the elongation, while “21 DPA” “25–28 DPA,” “30 DPA,” “35 DPA,” and “40 DPA” were defined for SCW stages. All four subsets were assumed to be representative of fiber overlapping development stages. Furthermore, we quantitated the transcripts through the StringTie assembler (Pertea et al., 2016) for each stage. These subsets were used to check the *in silico* expression pattern for downstream analysis.

### Functional Enrichment and *Cis*-Regulatory Elements Identification of Differently Clustered Modules

Gene ontology (GO) analysis of differentially regulated genes was carried out through the AgriGO database (Du et al., 2010). Significantly enriched, no-redundant GO Terms were visualized through the R package (R Core Team, 2013). Mapman analysis was carried out where data points in each bin were averaged, and the resulting *p*-value was adjusted to the Bonferroni. Motif enrichment analysis was carried out by the MEME suite for at most 10 motifs by using “-mod zoopsminw 4 –maxw 10” parameters for the DEGs and random datasets.

To check the regulatory elements in all six modules, promoter analysis was conducted through the PlantPAN3.0 database (Chow et al., 2019). We retrieved the genomic sequences from 1,000 bp upstream of TSS of each gene in all modules and scanned further for the identification of the *cis*-regulatory elements (CRE) binding site. The frequency binding site for CRE for each module was calculated as – number of TF binding sites in the promoter sequences of each module multiplied by the motif length, which is further divided by the total number of genes present in that module multiplied by 100.

### Identification and *in silico* Validation *of* Exclusively Expressed Transcripts in Fiber Tissue

We accessed the SRA accessions “SRP049496” data and mapped the filtered reads on the reference genome to quantitate the exclusively expressed transcripts level at 0 DPA and 5 DPA in the natural fiberless mutant. Additionally, to scrutinize the expression status of exclusively expressed transcripts during the domestication process, we retrieved the RNAseq data of TM-1, domesticated cotton and TX2094, wild cotton cultivar at 10 DPA and 20 DPA fiber samples from the SRA accession “SRP017061.” All the accessed SRA data were processed in a similar way by which the previously downloaded SRA data were processed.

### Assembly of Unmapped Reads

Approximately, 1 billion unmapped reads were *de novo* assembled independently by the trinity assembler (Haas et al., 2013) with a contig length of more than 300 bp. A total of 482,285 contigs were generated and blast with the nucleotide datasets to select the best hits. With >90% similarity on 80% of coverage length of the blast query, we selected 25 thousand contigs to cluster into Supertranscripts using Lace software (Davidson et al., 2017). Unmapped reads were further mapped on assembled supertranscripts. These supertranscripts were used as a reference for quantification of unmapped reads followed by the functional assignment by GO and Kyoto Encyclopedia of Genes and Genomes (KEGG) databases (Ogata et al., 1999).

### RNA Extraction and RT PCR Validation

Total RNA was isolated from cotton cultivar Coker-312 at different stages of ovules (−3 and 0 DPA), fibers (10, 21, and 30 DPA), and leaf using the SIGMA Spectrum™ total RNA kit according to the protocol of the manufacturer. DNase treatment was done using the Ambion TURBO™ DNase kit, and RNA integrity was checked by electrophoresis. About 2 μg of DNaseI treated total RNA of each developmental stage was taken for the first-strand cDNA synthesis using SuperscriptIII (Invitrogen). The PCR was performed on ABI 7500 Fast Real-Time PCR Machine (Applied Biosystems, United States) using Fast SYBR™ Green Master Mix (Applied Biosystems). Furthermore, the relative expression levels (fold change) were determined from analysis of relative gene expression data using real time quantitative PCR and the 2^(−ΔΔCT)^ method (Schmittgen and Livak, 2001). UBQ14 (Ghir_D10G001850.1) was used as an endogenous control for normalization. Statistical analysis was done on two biological and three technical replicates for each developmental stage wherein the error bar represents the standard error. The list of primers used in this study was provided in Supplementary File 3.

### Planting and Phenotyping of 100 Cotton Genotypes

Phenotyping of different fiber quality and fiber yield-related traits were carried out in the 100 naturally available cotton genotypes that were grown at two different locations (Hyderabad and Aurangabad) of India in the 2018 and 2019 growing seasons. All genotypes were planted in an experimental field with three replicates in April and were harvested in mid of October.

Thirty naturally opened bolls were randomly picked from each replicate for ginning processes. Fiber samples of 30- to 50-gram weight were prepared for fiber testing. First, the samples were conditioned at 25^0^C ± 2 temperature and 65% ± 2 relative humidity for 24 h. The preconditioned samples were put for the High Volume Instrument (HVI) test for different fiber parameters. Thus, the fiber length (mm), uniformity index (%), fiber strength (g/tex), fiber elongation (%), and micronaire (μg/inch) were recorded for the fiber quality traits. Fiber yield-related traits namely, boll number, boll weight (g), and cotton yield per plot (Kg) were also registered in our selected genotypes.

### Nanostring nCounter Gene Expression Validation

Fiber samples from 0, 6, and 19 DPA were checked for RNA integrity, while a Nanodrop spectrophotometer was used to access the quantification of the total RNA. A total of 150 ng of total RNA was added to a master mix that contained a hybridization buffer, a gene-specific pool of probes, reported tags, and universal capture tags and hybridized at 65°C for 16 h. Gene-specific probes and reporter tags were designed by NanoString Technologies Inc., and provided in Supplementary File 4. The good quality samples were transferred to a nCounter cartridge followed by loading onto the Nanostring nCounter Sprint Profiler for hybridization and immobilization. Scanning of cartridge had been done by nCounter digital analyzer. The NanoString Norm package (Waggott et al., 2012) in R was used for quality check and data analysis. The mean expression value of three endogenous control genes, *viz.*, EF1a-5, UBQ-14, Actin-4 was used for normalizing the nCounter read count.

## Results

### A Comprehensive Gene Expression Atlas of Cotton RNAseq for Extraction of Fiber-Specific Features

**FIGURE 1:**
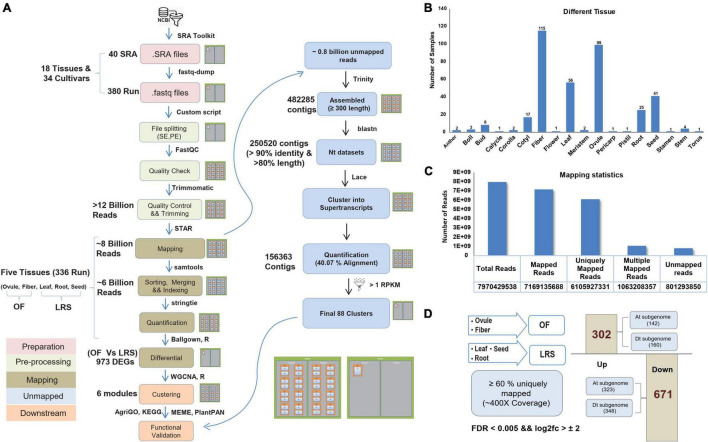
Processing of RNAseq data and their mapping statistics. **(A)** The complete pipeline was used to analyze the Sequence Read Archive (SRA) files on High Performance Computing (HPC) facility. **(B)** Different numbers of processed RNAseq samples of different eighteen cotton tissues. **(C)** Mapping statistics of five selected tissues, including the total number of reads, uniquely mapped reads, and unmapped reads. **(D)** Differential estimation of uniquely mapped reads of OF and LRS tissues. OF tissue spans ovule and fiber uniquely mapping reads, while LRS tissue consists of uniquely mapped reads of leaf, root, and seed tissues. About 302 genes were assigned as upregulated differentially expressed genes (DEGs), and 671 represented as the downregulated DEGs.

For the parallelization of billions of reads of 40 Sequence Read Archive (SRA) accessions (Supplementary File 1), we used multiple nodes on the HPC cluster and processed them with the same bioinformatics pipeline (Figure 1A). Such large data required a specific computation infrastructure to store, analyze, and retrieve the data. The SRA samples comprised of different cotton tissues, such as anther, boll, bud, calycle, corolla, cotyledon, fiber, flower, leaf, meristem, ovule, pericarp, pistil, root, seed, stamen, stem, and torus of the different cultivars (Supplementary Figure 1). We considered data only from five tissues (ovule, fiber, leaf, root, and seed) as those having the highest number of SRA samples (336 runs) (Figure 1B) to represent the fiber and non-fiber tissues. Out of approximately eight billion filtered reads, 6.1 billion reads were uniquely mapped to the cotton genome (Figure 1C) representing 400× coverage to ∼2.8 Gb genome size. Approximately 0.8 million unmapped reads were also processed independently and assembled into super-transcripts (Figure 1A). Total 88 super-transcripts were quantified having the Reads Per Kilobase Million (RPKM) value > 1 (Supplementary File 5). The GO and KEGG pathway analysis indicates their role in metabolic pathways and oxidative phosphorylation, as well as associated with nutrient reservoir and seed maturation activity (Supplementary Figure 2).

The primary aim of this study is to develop a comprehensive gene expression atlas to extract the fiber-specific features at different fiber development stages. We pulled uniquely mapped reads (Supplementary File 2) from the ovule and fiber tissues into one group and termed them as OF, while uniquely mapped reads from leaf, root, and seeds were pulled together and termed as LRS (Supplementary File 6). Differential expression analysis identified a total of 973 DEGs (FDR < 0.005 and log2fold change > 2) between OF and LRS, out of which 302 genes were upregulated and 671 genes were downregulated (Supplementary File 7). At the subgenome level, 142 and 160 genes were upregulated, whereas 323 and 348 genes were downregulated for cotton At and Dt subgenome, respectively (Figure 1D). The functional relevance of 302 upregulated DEGs for fiber development was validated by comparing their expression in the wild cotton (Xu142) and its fiberless mutant (Xu142 *fl*) at 0 DPA and 5 DPA fiber developmental stages. The expression of 302 upregulated DEGs was found significantly lower at 0 DPA and 5 DPA in Xu142 *fl* as compared to Xu142, substantiating their role in fiber development (Supplementary Figure 3).

### The Distinct *Cis*-Regulatory Sequence Governs the Fiber-Specific Expression of the At and Dt Genomes

**FIGURE 2:**
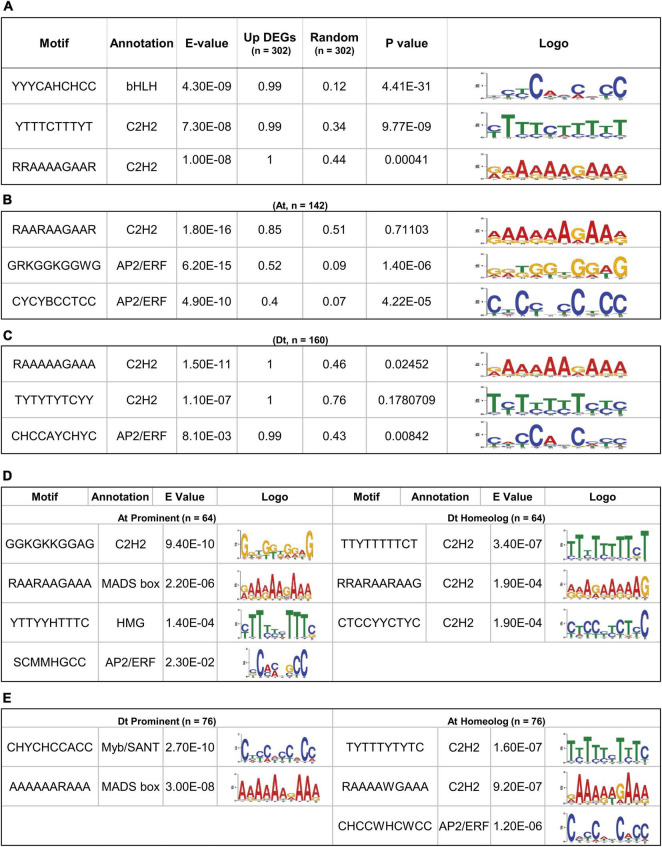
Significant motif enrichment analysis of upregulated DEGs with the random datasets and their homeolog. **(A)** The three enrichment motifs of 302 upregulated DEGs with the E-value < 0.05 and their significant frequency of occurrences (*P* < 0.05) with a similar number of random datasets. **(B)** Significantly enriched motif of 142 At-containing DEGs and **(C)** 160 Dt-containing DEGs with the random datasets. **(D)** Motif enrichment of 64 out 142, At prominent DEGs with their respective Dt homoeologous genes and **(E)** motif enrichment of 76 out of 160 Dt prominent DEGs with their respective At homoeologous genes.

The conserved motifs that could be responsible for regulating the fiber-specific expression of 302 upregulated DEGs were identified in the promoter sequences (1,000 bp upstream of TSS) using MEME suite (Bailey et al., 2015). The significance of identified motifs was ascertained by E-value in MEME and determining its significance (*p*-value) in a random-promoter dataset of a similar number. The MEME of the 302 gene’s promoter identified three statistically significant motifs, and their enrichment was also significant over the random dataset. The annotation of identified motifs indicates that the topmost significantly identified motif YYYCAHCHCC showed the consensus bHLH transcription factor (TF) binding site, while the other three motifs, *viz.*, YTTTCTTTYT and RRAAAAGAAR belong to the consensus C2H2 zinc finger TFs binding site (Figure 2A). We next identified conserved motifs belonging to At and Dt subgenome-related upregulated DEGs, separately, that identified three statistically significant motifs for each (Figures 2B,C). Interestingly, the topmost significant motif RAARAAGAAR showed the consensus C2H2 zinc finger TFs binding site in At subgenome (Figure 2B). The other two conserved motifs, *viz.*, GRKGGKGGWG and CYCYBCCTCC, belong to the consensus AP2/ERF TFs binding site, which were also significant with the random promoter dataset. The most significant motif, RAAAAAGAAA in Dt subgenome, was somewhat similar to the topmost identified motif in At subgenome and belongs to the consensus C2H2 zinc finger TFs binding site. The other two motifs, *viz.*, TYTYTYTCYY and CHCCAYCHC, belonging to consensus C2H2 and AP2/ERF TFs binding sites, were quite different from the motif identified in At subgenome (Figure 2B). In Dt subgenome-upregulated DEGs, RAAAAAGAAA and CHCCAYCHC motifs were also statistically significant over a similar number of random promoter datasets.

The differences in the conserved motifs identified for At and Dt subgenomes prompted us to correlate *cis*-regulatory element bias with the expression of At and Dt prominent genes. We thus identified 64 out of 142 At prominent and 76 out of 160 Dt prominent genes whose gene expression ratio with their respective homeologs was > 1 (Supplementary Files 8, 9). The 64 At prominent genes showed four statistically significant motifs, *viz*., GGKGKKGGAG (C2H2), RAARAAGAA (MADS-box), YTTYYHTTTC (HMG factors), and SCMMHGCC (AP2/ERF). However, Dt homeolog of these 64 At prominent genes showed three conserved motifs, *viz.*, TTYTTTTTCT, RRARAARAAG, and CTCCYYCTYC, belonging to the consensus C2H2 zinc finger TF binding site (Figure 2D). In the case of Dt prominent 76 genes, two statistically significant motifs, *viz.*, CHYCHCCACC (Myb/SANT) and AAAAARAAA (MADS-box), were very distinct from the consensus motif identified in their At homeolog, namely TYTTTYTYTC (C2H2), RAAAAWGAAA (C2H2), and CHCCWHCWCC (AP2/ERF) (Figure 2E).

### Clustering of Differentially Expressed Genes at Different Fiber Developmental Stages

**FIGURE 3:**
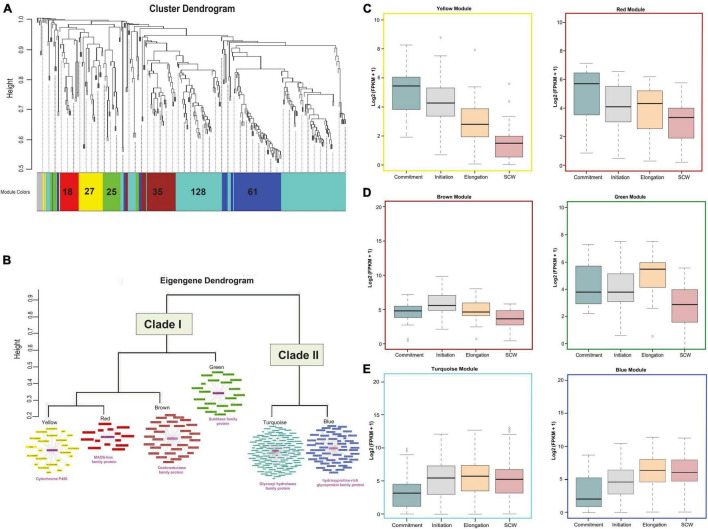
Downstream analysis of upregulated DEGs. **(A)** Module-specific hierarchical clustering of upregulated genes (302 genes with the log 2fc > 2 and FDR < 0.005) in OF tissue. Different colors represent different modules, and the encrypted number indicates the total number of genes present in those modules. **(B)** Eigengene dendrogram of six modules that were grouped into two major clades. The co-expression behavior of genes present in each module was visualized through a co-expression network in which the magenta color shows the hub gene. The putative annotation of the hub gene was mentioned below the clade. Functional characterizations of different modules at Commitment, Initiation, Elongation, and secondary cell wall (SCW) fiber developmental stages for **(C)** yellow and red modules, **(D)** brown and green modules, and **(E)** turquoise and blue modules.

Differential expression analysis was further considered for the clustering based on the weighted gene co-expression network analysis. Out of 302 upregulated genes, 8 genes were excluded as outliers, and the remaining 294 genes were clustered with the soft thresholding power of 10 to construct a block-wise module (Figure 3A). As a result of this, we obtained six modules represented in red (18 genes), yellow (27 genes), green (25 genes), brown (35 genes), turquoise (128 genes), and blue (61 genes) colored schemes. Based on eigengene relationships, block-wise clustered six modules were further grouped into two clades, Clade I representing red and yellow modules with brown and green module outliers and Clade II consisting of blue and turquoise modules (Figure 3B). The expression profiling of genes belonging to Clade I (especially for red and yellow modules) reflects a higher expression level at the fiber commitment stage (Figure 3C), and Clade II (blue and turquoise) showed higher expression at fiber elongation and SCW stages (Figure 3E). Cytochrome P450 gene represented as a hub gene for the yellow module, whereas the red module containing genes was connected to the MADS-box family protein (Figure 3B). In the outlier modules of Clade I, brown and green modules also showed their relatively higher expression at the initiation and elongation stages (Figure 3D) with the central key players as glucose-methanol-choline oxidoreductase family protein and subtilase family protein, respectively. Glycosyl hydrolases family protein and hydroxyproline-rich glycoprotein family were the central key players for turquoise and blue modules of Clade II, respectively (Figure 3B), which represent the entire module. The downregulated genes were also clustered into four major modules (Supplementary Figure 4) and have a functional role in photosystem-related pathways (Supplementary Figure 5) and in response to abiotic and temperature stimuli (Supplementary Figure 6).

### Functional Validation of Upregulated Clustered Modules and Their *Cis*-Regulatory Enrichment

**FIGURE 4:**
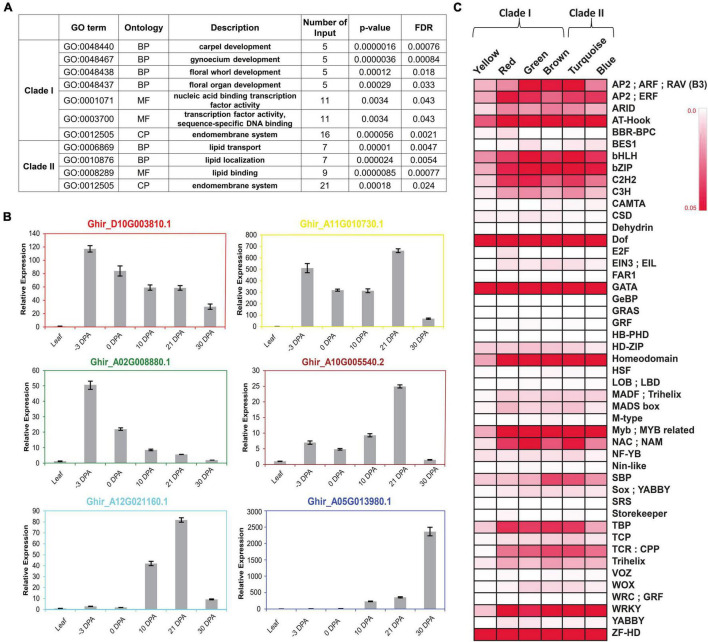
Functional enrichment of each clustered module and clade. **(A)** *Arabidopsis* based significant gene ontology (GO) enrichment of two major clades, *viz.*, Clade I and Clade II. False Discovery Rate (FDR) value < 0.05 was used for checking the significance level of each GO Term. **(B)** Real-time expression analysis of hub gene of all six modules. Hub genes are the representative of each module that confirmed their expression behavior through real-time expression analyses in leaf tissues, –3 DPA, 0 DPA, 10 DPA, 21 DPA, and 30 DPA of fiber developmental stages. **(C)** Binding frequency of *cis*-regulatory elements in the promoter region of genes is present in all six modules, *viz.*, yellow, red, green, brown, turquoise, and blue. Color code ranges from 0 to 0.05; zero represents the lowest binding frequency of *cis*-regulatory element (CRE), while 0.05 depicted the higher binding frequency of CRE over the promoter regions.

To check the functional enrichment, GO analysis was carried out for each clade, individually suggesting the involvement of Clade I in different reproductive organ development and in TF activities, whereas Clade II was significantly enriched for the lipid transport, localization, and lipid-binding processes (Figure 4A). Additionally, the central key player of each identified module in the form of a hub gene was validated through the real-time expression analyses (Figure 4B). The relatively higher expression level of hub genes at −3 DPA for red, yellow, and green modules, suggesting the gene present in the respective modules, was involved and co-expressed for the fiber commitment process. The dual expression of cytochrome P450 (Ghir_A11G0010730.1) was validated where it was relatively expressed at fiber commitment (−3 DPA) as well as in SCW stages (21 DPA). The hub genes of the other three modules namely, brown, turquoise, and blue modules were validated for the fiber elongation and SCW stages, respectively.

The different expression levels of each module at different fiber developmental stages might be regulated by the various CREs present in their promoter region. We identified the binding frequency of the CRE in 1,000 bp upstream of Transcriptional Start Site (TSS) using the PlantPAN3.0 database (Chow et al., 2019). The higher binding frequency of CRE in each module represents its pivotal role to regulate the expression of its respective genes. A total of 47 TF binding sites were identified in which Dof, GATA, and ZF-HD TFs-binding frequency were very prominent in all six modules (Figure 4C). Homeodomain, MYB/Myb related, NAC, and WRKY TFs-binding sites frequency were comparatively higher in all modules except for yellow. Four modules, *viz.*, turquoise, red, green, and brown modules have high TATA Binding Protein (TBP) frequency while low in blue and yellow modules. Squamosa promoter binding protein (SBP) has a higher binding frequency in turquoise and brown modules, which was designated as an elongation-specific module (Figures 3E, 4C).

### Module-Specific Pathway Enrichment of Upregulated Genes in Different Fiber Developmental Stages

**FIGURE 5:**
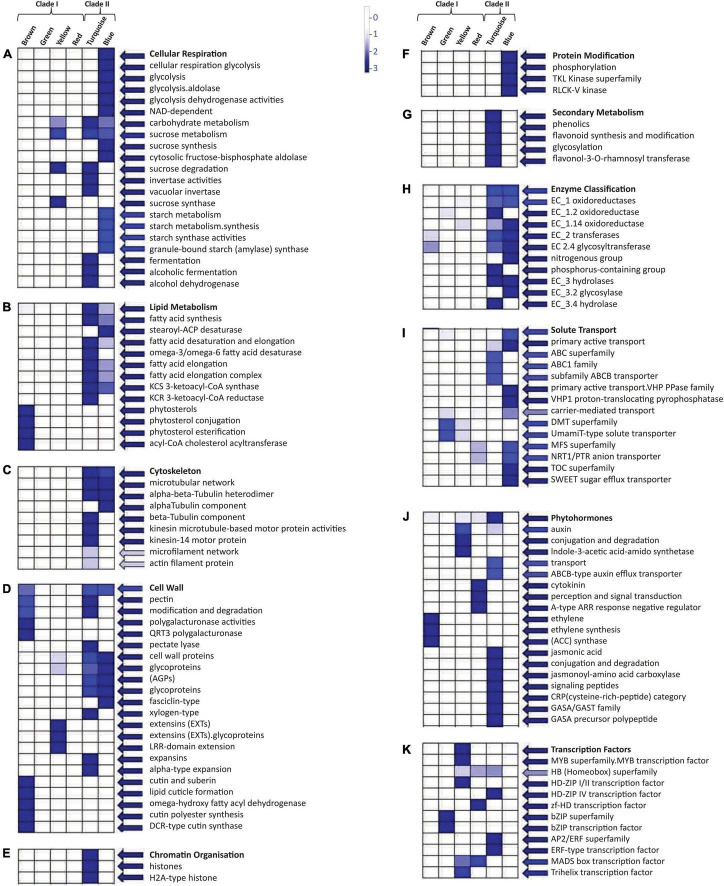
Mapman based significant functional classification of two different clades. These two clades consist of six modules that were involved in different pathways: **(A)** cellular respiration and carbohydrate metabolism, **(B)** lipid metabolism, **(C)** cytoskeleton molecules, **(D)** cell wall-related functioning, **(E)** chromatin organization, **(F)** protein modification, **(G)** secondary metabolism pathway, **(H)** enzymatic action, **(I)** solute transportation, **(J)** phytohormone, and **(K)** transcription factors. BINs color code ranging from 0 to 3 with the vertical bar that represents the log2 expression value of each module.

Mapman pathway analysis revealed that the modules present in Clade II were involved in cellular respiration as well as in carbohydrate, sucrose, starch, and lipid metabolism (Figures 5A,B). Thus, it was associated with cell wall formation through the upregulated expression of pectin and many glycoproteins (Figure 5D). Many cytoskeletons-related components like alpha, beta-tubulin, and actin filament were also upregulated in Clade II (Figure 5C) with oxidoreductase, transferases, and hydrolases like enzymatic activities (Figure 5H). The turquoise module of Clade II was involved in chromatin modification (Figure 5E) and the secondary metabolism pathway (Figure 5G), whereas the blue module assists protein modification and phosphorylation *via* tyrosine kinases (TKL) (Figure 5F). The upregulated genes of these two modules were involved in solute transport through the involvement of ABC, VHP PPase, MFS, and TOC transporter (Figure 5I) while DMT and MFS transporters were only upregulated in the Clade I module. In the presence of the extensin gene (Figure 5D), the yellow module is involved in sucrose degradation and thus in cell wall formation (Figure 5A). The auxin hormone pathway was also enriched in the yellow module (Figure 5J), along with the MYB superfamily TFs (Figure 5K). Other TFs like the homeobox superfamily, MADS-box, and trihelix were also aligned with this module, whereas bZIP TFs were upregulated for the green module and AP2/ERF and homeobox superfamily for the turquoise module. Moreover, the brown module’s genes facilitated the cuticle formation, phytosterol conjugation, and pectin modification (Figure 5D) with ethylene hormone responsive genes, while the red module manifested the cytokinin perception and signal transduction (Figure 5J).

### The Exclusively Expressed Gene Variants in Cotton Fiber Development

Besides 302 upregulated genes in OF, we identified 20 transcripts that expressed exclusively in OF as compared to LRS. Expression of these transcripts was very significant as we processed approximately ∼ 400× uniquely mapped read coverage to the cotton reference genome. It is very interesting to note that the expression of these gene variants was very specific (Table 1) to the OF tissues as compared to their other variants, which showed somewhat higher expression in non-fiber tissue (LRS). All these gene variants belong to the ELO gene, PDF2, WRKY, and bHLH TFs, several enzymes (glycerol-3 phosphatase dehydrogenase and benzoquinone reductase), and many hypothetical proteins. The real-time PCR analyses represent the typical expression as determined by *in silico* transcriptome analysis (Supplementary Figure 7). In real-time expression analysis, hypothetical protein (Ghir_D10G025770.3) expressed at −3 DPA and 0 DPA and serine threonine-protein kinase transcript (Ghir_D05G003410.3) shows significant expression at the elongation stage with null expression value in the leaf tissue.

**TABLE 1:** Detail information of Exclusively Expressed Transcripts (EETs), including their *Arabidopsis*-based annotation and log2 (FPKM + 1) value in OF and LRS tissues.

Gene	Annotation	Transcripts	OF	LRS
	
			Log2 (FPKM + 1)	
Ghir_D01G000100	**ELO/SUR4 family**	Ghir_D01G000100.1	8.247181314	0
		Ghir_D01G000100.2	5.849377026	1.3980718
Ghir_A10G001030	**HD zipper protein protodermal factor 2**	***Ghir_A10G001030.1***	0.285908775	0.0775192
		***Ghir_A10G001030.2***	1.082713488	1.0809891
		Ghir_A10G001030.3	2.907309878	0
Ghir_D09G019020	**NAC domain-containing protein 62-like**	Ghir_D09G019020.1	2.640708144	3.0826249
		Ghir_D09G019020.2	1.314970249	1.8542175
		Ghir_D09G019020.3	1.44367189	1.9737428
		Ghir_D09G019020.4	2.6234633	0
Ghir_A09G012990	**glycerol-3-phosphate dehydrogenase**	***Ghir_A09G012990.1***	0.087107046	0.4593403
		***Ghir_A09G012990.2***	1.204346712	0.4166236
		***Ghir_A09G012990.3***	0.875061288	0.8913321
		***Ghir_A09G012990.4***	2.617127776	0
Ghir_D01G006540	**Benzoquinone reductase**	Ghir_D01G006540.1	6.268913584	3.9051894
		Ghir_D01G006540.2	0.999345589	0.690696
		Ghir_D01G006540.3	1.020565494	0
Ghir_D10G025770	**Hypothetical protein**	Ghir_D10G025770.1	0.92436369	0.0527247
		Ghir_D10G025770.2	1.094223231	0.2255871
		Ghir_D10G025770.3	0.534289745	0
		Ghir_D10G025770.4	1.084769108	0.1948385
Ghir_A05G038240	**WRKY transcription factor 1**	***Ghir_A05G038240.1***	4.778754111	1.9936975
		***Ghir_A05G038240.2***	2.929963278	0.137295
		***Ghir_A05G038240.3***	0.634027231	0.033738
		***Ghir_A05G038240.4***	0.940046443	0.0363838
		***Ghir_A05G038240.5***	1.671006617	0.0342636
		***Ghir_A05G038240.6***	0.451709621	0
Ghir_D05G003410	**Serine-threonine protein kinase**	Ghir_D05G003410.1	2.98741965	0.6356541
		Ghir_D05G003410.2	0.303920993	0.0050133
		Ghir_D05G003410.3	0.280408817	0
		Ghir_D05G003410.4	0.096578968	0.0057262
Ghir_D11G022110	**bHLH61 like transcription factor**	***Ghir_D11G022110.1***	0.235100837	0
		***Ghir_D11G022110.2***	2.182144669	1.0477708
		Ghir_D11G022110.3	1.617503963	0.7769445
Ghir_A08G022470	**WVD1 like 1 isoform**	Ghir_A08G022470.1	1.282175262	0.2576479
		***Ghir_A08G022470.2***	0.095796175	0.2643491
		***Ghir_A08G022470.3***	0.099241334	0.8651611
		Ghir_A08G022470.4	0.589878515	0.8751675
		Ghir_A08G022470.5	0.227819923	0
		***Ghir_A08G022470.6***	0.202128056	0
		Ghir_A08G022470.7	1.320330436	0.0007414
Ghir_A11G025670	**Hypothetical protein**	***Ghir_A11G025670.1***	2.417842256	0.7236608
		Ghir_A11G025670.2	0.188235059	0
		***Ghir_A11G025670.3***	0.238984911	0.3046851
Ghir_D01G005620	**Hypothetical protein**	Ghir_D01G005620.1	2.047156551	1.1493537
		Ghir_D01G005620.2	0.153845536	0
		Ghir_D01G005620.3	0.888239423	0.7282053
Ghir_D01G020410	**Hypothetical protein**	Ghir_D01G020410.1	0.073779111	0
Ghir_D04G015610	**bifunctional epoxide hydrolase 2 like gene**	***Ghir_D04G015610.1***	0.053649379	0
		***Ghir_D04G015610.2***	5.945628677	1.2868179
Ghir_A09G014480	**Hypothetical protein**	***Ghir_A09G014480.1***	0.0413986	0
		***Ghir_A09G014480.2***	1.144999148	0.6043513
Ghir_A12G003890	**Hypothetical protein**	Ghir_A12G003890.1	0.011841959	0
		Ghir_A12G003890.2	4.091971001	0.926886
Ghir_A09G013880	**Hypothetical protein**	Ghir_A09G013880.1	0.740766125	0.2872205
		Ghir_A09G013880.2	3.114859556	1.437379
		Ghir_A09G013880.3	0.781812072	0.3974635
		Ghir_A09G013880.4	0.007735155	0
Ghir_D12G002480	**LAG1 longevity assurance homolg2-like**	Ghir_D12G002480.1	0.001137838	0
		Ghir_D12G002480.2	2.875775739	0.410619
Ghir_D05G007320	**Hypothetical protein**	Ghir_D05G007320.1		0
		Ghir_D05G007320.2	7.705065032	3.6233814
		Ghir_D05G007320.3	0.616319112	0.0705858

*Identified EETs were highlighted with the olive green-colored cell whose expression was null in LRS tissue. Some identified transcripts have identical nucleotide sequences, which were represented by the bold and italic fonts in the transcripts column.*

### Evolution of Exclusively Expressed Transcripts and Their Validation in 100 Cotton Genotypes

**FIGURE 6:**
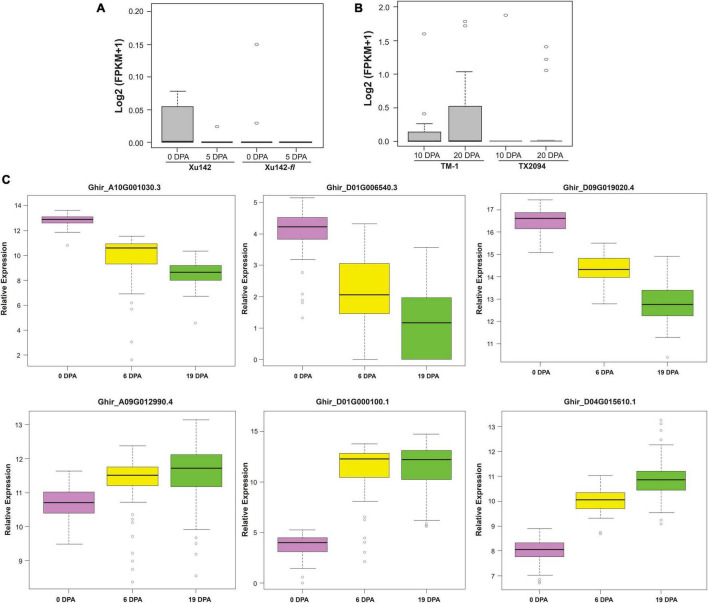
Validations of Exclusively Expressed Transcripts (EETs) through RNAseq expression profiling and nCounter assay. **(A)** the *in silico* expression pattern of twenty EETs for the fiber-specific pattern in wild cotton (Xu142) and *fuzzless* natural mutant (Xu142*fl*) at 0 DPA and 5 DPA as well as **(B)** for the domestication process in modern-day cotton (TM-1) and wild cotton (TX2094) at 10 and 20 fiber developmental stages. **(C)** Nanostring based nCounter gene expression assay of six selected transcripts at 0, 6, and 19 DPA fiber developmental stages in 100 cotton genotypes growing at the 2018 growing season at Hyderabad city of India.

Exclusive expressed transcripts (EETs) were very specific to the fiber structures, so to substantiate their importance in the cotton genome, we checked the collective expression of these transcripts in fiberless mutant, Xu142*fl*, and wild cotton cultivar, Xu142 (Figure 6A). In fiberless mutant cotton, these transcripts show null expression at 0 DPA and 5 DPA fiber developmental stages, indicating their potential role in various stages of fiber development, especially at the initiation stage. We also checked the expression profiling of these 20 gene variants in wild cotton, TX2094, and domesticated cotton, TM-1 (Figure 6B). The cumulative expression of 20 EETs was significantly low at 10 DPA and 20 DPA in wild variety than the domesticated variety. These observations further corroborate the functional importance of these genes in the cotton fiber developmental stages during the different evolutionary processes.

We selected five highly expressed transcripts [log2 (FPKM + 1) > 1], *viz.*, the ELO/SUR4 family, PDF2, NAC domain-containing protein 62 like, glycerol-3-phosphate dehydrogenase, benzoquinone reductase, and one low expressed transcript [log2 (FPKM + 1) < 0.1] bifunctional epoxide hydrolase 2 for expression and correlation study in 100 cotton genotypes (Supplementary File 10). Different fiber quality and fiber-yield traits were estimated for 2 consecutive years, *viz.* 2018 at Hyderabad, 2019 at Aurangabad and Hyderabad. Aurangabad and Hyderabad are two different cities of Maharashtra and Telangana states, respectively, which were reported as the leading states in cotton acreage in India (Nagrale et al., 2020). The phenotypic relevance of these fiber quality traits was highly correlated with each other in two different seasons (2018 and 2019) (Supplementary Figure 8) and different locations (Hyderabad and Aurangabad) (Supplementary Figure 9). Expression of selected exclusively expressed transcripts was further examined using nanostring nCounter technology at 0 DPA, 6 DPA, and 19 DPA in 100 genotypes of cotton that were grown in 2018 at Hyderabad city of India (Figure 6C). The nCounter expression analysis confirmed that PDF2, NAC domain-containing protein 62 like, and benzoquinone reductase were highly expressed at 0 DPA, while ELO/SUR4, glycerol-3-phosphate dehydrogenase, and bifunctional epoxide hydrolase 2 like gene were significantly highly expressed at 6 DPA and 19 DPA. The expression of exclusive transcripts observed in the nCounter assay confirmed their expression pattern, which was obtained through *in silico* analysis and thus further validated our results.

### The Expression of Exclusively Expressed Transcripts Correlates With the Quality Parameters of Cotton Fiber

**FIGURE 7:**
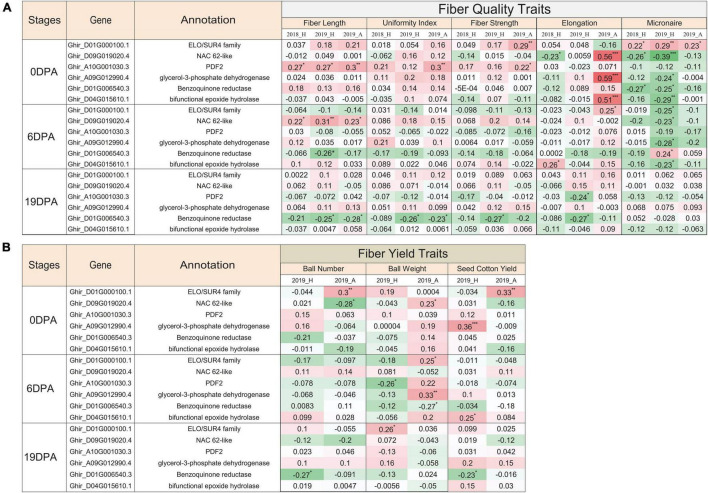
Functional validation of EETs for different fiber-related traits. Pearson correlation coefficient of nanostring nCounter gene expression value of 100 cotton genotypes grown in 2018 at Hyderabad city with phenotypic characteristics of different **(A)** fiber quality-related traits and **(B)** fiber yield-related traits for three different conditions, viz., 2018_H, 2019_H, and 2019_A. 2018 and 2019 represented the two consecutive growing seasons, while H and A depicted as Hyderabad and Aurangabad cities of India, respectively. The green-colored cell represented the positive correlation, whereas the red-colored cell represented the negative correlation value. Significant correlation was denoted with the asterisk mask (*) in which *, **, *** used for the *p*-value ≤ 0.05, ≤ 0.01, and ≤ 0.005, respectively.

In correlation and statistical analysis of nanostring expression and fiber quality traits in 100 genotypes, the ELO/SUR4 family gene variant exhibits a significant positive correlation with fiber strength, micronaire, boll number, and cotton yield at 0 DPA (Figures 7A,B). This transcript also correlated with elongation, micronaire, and boll weight traits at 6 DPA and only with the boll weight at 19 DPA. An unlikely significant negative correlation was seen with the micronaire at 6 DPA in 2019 at Hyderabad city. The NAC domain-containing protein 62 like exhibits a negative significant correlation with the micronaire trait at 0 DPA and 6 DPA, while the positive correlation with fiber length at 6 DPA in 2 consecutive years (2018, 2019) as well as in two different locations (Hyderabad, Aurangabad). Contrasting behavior of the NAC domain gene was observed in 2018_H and 2019_A data at 0 DPA where we observed significant negative and positive correlations, respectively, with the fiber elongation trait. At 0 DPA, HD zipper protein protodermal factor 2 transcripts showed a significant positive correlation with the fiber length, uniformity index, and fiber strength, while a significant negative correlation with boll weight at 6 DPA for 2019_H. Glycerol-3-phosphate dehydrogenase and bifunctional epoxide hydrolase 2-like transcripts showed significant positive and negative correlations with elongation and micronaire, respectively, at 0 DPA (Figure 7A). The bifunctional epoxide hydrolase 2-like transcript was also positively correlated with the fiber elongation at 0 DPA and 6 DPA and cotton yield at 6 DPA. The benzoquinone reductase transcript displayed a significant negative correlation with the micronaire at 0 DPA, fiber length and boll weight at 6 DPA, and fiber length, uniformity index, fiber strength, elongation, boll number, and cotton yield at 19 DPA (Figures 7A,B). All selected transcripts show the significant negative correlation with micronaire at 0 DPA and 6 DPA, exceptional ELO at 0 DPA, and benzoquinone reductase at 6 DPA. All the mentioned correlations were significantly affected by the fiber-related traits either positively or negatively.

### “CottonExpress-omics” Database

CottonExpress-omics represents the comprehensive gene expression atlas of cotton transcriptome data to unravel the fiber-specific expression features at different developmental stages and is available at the http://14.139.63.169/cottonexpressomics. The database aids researchers with a comprehensive account of fiber-specific DEGs, EETs, clustered modules, and conserved motifs that were mined in the present study. Cotton community can obtain the expression value of the gene of interest from diverse cotton cultivars or for different fiber developmental stages under the “FPKM” subsection of the “Mapping Statistics” menu. The fold change expression value of OF vs. LRS and the annotation of different members of clustered modules can also be searched by users under the “Downstream” menu. Thus, this database provides elaborative information of different fiber-specific features under one roof that will be helpful for the cotton researchers and breeders for cotton fiber improvement.

## Discussion

The cotton genome was refined over the years by different independent groups to get the well-annotated genes and unravel the evolution dynamics for fiber-specific features (Yang et al., 2020). Additionally, to understand the molecular and biological functions of genes involved in cotton fiber growth and development, the multi-omics approach was also taken into consideration (Pang et al., 2009; Rai et al., 2013; Song et al., 2015, 2017; Zou et al., 2016; Kumar et al., 2018; Ayubov et al., 2019; Li et al., 2019). In recent years, many researchers have reannotated the cotton genome sequence by third and fourth generation sequencing techniques to get the high contiguity genome (Pan et al., 2020). Prior to cotton genome sequence being made available, several transcriptome data (Wang et al., 2010; Nigam et al., 2014; Peng et al., 2014; Cheng et al., 2016; Parekh et al., 2018) were generated that would not have been mapped properly due to the unavailability of a well-furnished reference genome. Recent efforts have been carried out for the identification of important molecular signatures for fiber-related traits, in which around fivefold to sevenfold coverage transcriptome data were generated and mapped on the draft genome of cotton (Fang et al., 2017; Ma et al., 2018). The low coverage on the draft genome may not be used for the identification of low expressed genes and its variants that might be crucial for fiber development. Similarly, in an eQTL identification study, only 10 billion reads were used for 251 cotton accessions (Li et al., 2020) and only focused on the initiation of secondary cell wall synthesis at 15 DPA. Thus far, no studies have been available that used high coverage cotton transcriptome data on the newly assembled genome (Hu et al., 2019; Wang et al., 2019; Yang et al., 2019; Chen et al., 2020; Huang et al., 2020) for all fiber developmental stages. In our current study, we elucidated many fiber-specific features for different fiber developmental stages, categorizing them into commitment, initiation, elongation, and SCW synthesis stages where we processed and reanalyzed the 356 publicly available cotton transcriptome data on the HPC cluster. The HPC cluster accomplished the computation requirement for such a large dataset, which was processed through a similar bioinformatics pipeline in the batch script. The high-coverage data helped us in the identification of important fiber features that were not reported yet for different fiber developmental stages, which subsequently correlated with the different fiber-related traits.

Motif enrichment analysis of 302 differentially expressed fiber-specific genes revealed a significant enrichment of *cis*-regulatory motifs belonging to bHLH and C2H2 zinc finger TFs (Figure 2A). The TFs that interact with these two motifs are required for different transcriptional regulations at fiber initiation and elongation stages (Wang L. et al., 2021; Wang N. N. et al., 2021; Wu et al., 2021). However, in At subgenome (142) and Dt subgenome specific (160) DEGs, instead of bHLH, AP2/ERF TF showed significant motif enrichment over the random datasets (Figures 2A,C), which was previously reported to control the fiber length (Mittal et al., 2015). The enrichment of distinct motifs for At/Dt subgenome indicates the distinctness of the transcriptional regulatory apparatus that interacts by which they are expressed specifically during cotton fiber development. Many reports also indicated the similar gene expression biasness at the subgenome level during fiber development and its connection with the *cis*-regulatory motifs (Song et al., 2017; Zhao et al., 2018). Thus, we were also keen to link the biased expression between At and Dt subgenomes to *cis*-regulatory motifs in our analysis (Figures 2D,E). The MEME revealed a very distinct set of motifs that were enriched in 64 At and 76 Dt prominent genes corresponding to their homeologs, which reassure the distinct regulatory mechanisms for fiber-specific gene expression. Taken together, our motif analysis indicates fiber-specific DEGs expression that was governed by the different *cis*-regulatory mechanisms involving distinct sets of TFs. Thus, it will be interesting to explore further how the coordination between At and Dt subgenomes operates transcriptionally to achieve fiber specificity.

The comprehensive clustering of fiber-specific genes based on their expression resulted in six distinct modules, which are further grouped into two prominent clades to represent the stage-specific expression (Figure 3). Clade I significantly represents the commitment-specific module involved in different organ developments, while Clade II module involved in the fiber cell elongation and maturation through lipid metabolism processes (Figure 4A). The yellow module’s genes were annotated as MYB, HD-ZIP, MADS, and Trihelix TFs (Figure 5K and Supplementary Figure 4). The role of these TFs like MYB/MYB like (MacHado et al., 2009; Walford et al., 2011) and homeodomain leucine zipper gene (Walford et al., 2012) in the fiber initials further affirmed the specificity of this module for commitment-specific expression. Interestingly, although the expression of cell fate-determining factors - MYB and HD-ZIP is significantly high in the yellow module; however, promoter analysis indicates the poor enrichment of their binding site in the promoter of the same module (Figure 4C). This indicates that the expression of MYB and HD-ZIP is governed by different regulators at the commitment stage, but they regulate the expression of genes of other modules as represented through CRE frequency in the promoter regions (Figure 4C). Furthermore, the significance of the yellow module is substantiated by the presence of genes belonging to the auxin and indole-3-acetic acid amido synthase pathway whose role was well established for cell fate determination (Zhang et al., 2011; Xu et al., 2019). The role of auxin efflux and influx is well documented in the fiber initials at −1 DPA (Petrášek et al., 2006; Zhang et al., 2017), and fiber protrudes through the upregulation of genes belonging to extension-related glycoproteins. Another module of Clade I, *viz.*, red module, shows the upregulation of the cytokinin and cytokinin perception and signal transduction genes, including A type ARR negative regulator (Figure 5J). Thus, fiber development may follow a cascade where the antagonistic behavior of auxin and cytokinin (Zeng et al., 2019) has been maintained by the upregulation of A-type ARRs. The functional significance of genes identified in each module was also ascertained since many genes fall in the genic and intergenic regions that were associated with several fiber-related traits as shown in previously identified genome-wide association study (Supplementary Table 1; Ma et al., 2018).

High-coverage transcriptome data were used to identify the full sequence diversity of the genome, including lowly expressed transcripts (Fu et al., 2014). In the present study, analysis of approximately six million uniquely mapped reads leads to identify 20 EETs which showed unique fiber specific expression. The importance of exclusivity of these transcripts in fiber tissue needs to be explored further; however, tissue-specific variants have been shown to regulate the flowering at different developmental stages in *Arabidopsis* (Rosloski et al., 2013; Ghelli et al., 2018). The tissue-specific expression of variants may be due to the presence of tissue-specific promoter architecture, which requires a tightly regulated transcriptional machinery (Srivastava et al., 2018; Das and Bansal, 2019; Schmitz et al., 2021). During the domestication of cultivated cotton species, these transcripts might be positively selected in modern cotton for fiber-specific features (Figures 6A,B). The comprehensive expression analysis of EETs usisng nCounter gene expression assay involving 100 genotypes and three development stages (0 DPA, 6 DPA, and 19 DPA) confirmed the different stage-specific expressions of EETs (Figure 6C) that correlated with different fiber-related traits (Figure 7). The significant correlations further ascertain the importance of EETs in cotton fiber development identified in our study.

One of the tested EETs, PROTODERMAL FACTOR 2 (Ghir_A10G001030.3), significantly correlated with the fiber length (Figure 7). This was consistent with the previous report wherein the role of PROTODERMAL FACTOR 1 and its mechanism in fiber development was well documented (Deng et al., 2012). Yet another EET that belongs to the NAC family gene correlates with the fiber length (Figure 7A) and has been documented for positive and negative regulations at different developmental stages (Shang et al., 2016; Zhang et al., 2018; Sun et al., 2020). ELO transcript (Ghir_D01G000100.1) also correlates positively with the micronaire at 0 DPA that putatively involved in the synthesis of very long-chain fatty acids (VLCFs) (Qin et al., 2007a; Zhang et al., 2015), which ultimately acts as a precursor for the sphingolipid biosynthesis to promote cotton fiber elongation (Qin et al., 2007a,b). Our nCounter gene expression suggests that ELO’s expression initiated early at 0 DPA; thus, it is very interesting to validate further how the early expression of ELO influences the micronaire trait. Our correlation study established an inverse relationship between fiber elongation and micronaire, which may be crucial for sustaining the yarn strength (Mathangadeera et al., 2020) by combining properties of fiber length, density, and maturity (Long et al., 2013).

Thus, our extensive and comprehensive reanalyzing of RNAseq resulted in the identification of several new genes whose expression might be crucial for fiber development, which was validated through the different approaches. Data mining identifies several important features, which were supported by the previous results and thus substantiates our findings. All the associated result of this study was systemized in the “CottonExpress-omics” database for the cotton researchers and breeders that will help the cotton community to quickly identify genes that are expressed at different developmental stages.

## Data Availability Statement

The datasets presented in this study can be found in online repositories. The names of the repository/repositories and accession number(s) can be found in the article/Supplementary Material.

## Author Contributions

PP and SS conceptualized the study and wrote the manuscript. PP performed all related analyses. PP and SB conceived the web-portal design. SA and PP executed the web-portal design. SB and SS helped in data interpretation. RV provided the RNA samples for qRTPCR. UK validated the relative expression results. DM and PB provided phenotypic data of 100 genotypes of cotton. AK helped in nCounter analysis. All authors contributed to the article and approved the submitted version.

## Conflict of Interest

The CSIR-NBRI has an official collaboration with Tierra Seed Science Pvt. Ltd., for cotton research where DM and PB are employed. The remaining authors declare that the research was conducted in the absence of any commercial or financial relationships that could be construed as a potential conflict of interest.

## Publisher’s Note

All claims expressed in this article are solely those of the authors and do not necessarily represent those of their affiliated organizations, or those of the publisher, the editors and the reviewers. Any product that may be evaluated in this article, or claim that may be made by its manufacturer, is not guaranteed or endorsed by the publisher.
