# Supplementary material for: Transcriptional Landscape of Cotton Fiber Development and Its Alliance With Fiber-Associated Traits
Source: Front Plant Sci. 2022 Feb 24;13:811655. doi: 10.3389/fpls.2022.811655 (PMC8908376; doi:10.3389/fpls.2022.811655)
Supplement: Supplementary file 1 [file Data_Sheet_1.PDF]

Supplemental Table 1 Mapping of genes present in all six modules on previously published GWAS study

	GWAS	NAU_ID	SNP Position	Annotation	Ref	Alt	Ghir_ID	Chr	Start	End	Module	Annotation
Fiber Quality Traits	Fiber Length	Gh_A03G0838	A03_45369489	intergenic	A	T	Ghir_A03G010930.1	A03	53753347	53755749	Turquoise	polygalacturonase 2
		Gh_D11G1879	D11_21969284	intergenic	A	G	Ghir_D11G019790.2	D11	22495763	22497431	Yellow	homeobox protein 40
	Micronaire	Gh_D11G1879	D11_22051275	intergenic	A	G	Ghir_D11G019790.2	D11	22495763	22497431	Yellow	homeobox protein 40
	Elongation	Gh_D06G2306	scaffold4090_D06_56828	intronic	G	A	Ghir_A06G002250.1	A06	2502629	2504685	Turquoise	Glycosyl hydrolase family 35 protein
		Gh_D08G0148	D08_1308194	intergenic	T	C	Ghir_D08G001670.1	D08	1371505	1374066	Turquoise	Bifunctional inhibitor/lipid-transfer protein/seed storage 2S albumin superfamily protein
	Fiber length Uniformity	Gh_A09G0848	A09_55955687	intergenic	G	A	Ghir_A09G009800.1	A09	60902447	60904614	Turquoise	fatty acid desaturase 3
		Gh_D11G1879	D11_22051275	intergenic	A	G	Ghir_D11G019790.2	D11	22495763	22497431	Yellow	homeobox protein 40
	Fiber maturity	Gh_D05G0626	D05_5010329	intergenic	C	G	Ghir_D05G006440.1	D05	5172811	5174685	Brown	Peroxidase superfamily protein
		Gh_A08G0877	A08_50415585	intronic	C	G	Ghir_A08G011050.1	A08	72389055	72391573	Brown	HXXXD-type acyl-transferase family protein
		Gh_A08G1098	A08_77437901	intergenic	C	T	Ghir_D08G013970.1	D08	48250398	48253303	Yellow	cytochrome P450, family 78, subfamily A
		Gh_D11G2930	D11_59745512	intergenic	T	A	Ghir_D11G032280.1	D11	66692382	66693310	Green	Plant invertase/pectin methylesterase inhibitor superfamily protein
		Gh_D12G1304	D12_41367998	intergenic	G	A	Ghir_D12G014120.1	D12	44456678	44457490	Turquoise	Protein of unknown function, DUF538
Fiber Yeild Traits	Spinning index	Gh_D11G1879	D11_22038558	intergenic	G	T	Ghir_D11G019790.2	D11	22495763	22497431	Yellow	homeobox protein 40
	Lint Percentage	Gh_A08G0877	A08_50374894	downstream	G	C	Ghir_A08G011050.1	A08	72389055	72391573	Brown	HXXXD-type acyl-transferase family protein
		Gh_D01G0856	D01_14049204	intergenic	T	C	Ghir_D01G009870.1	D01	15432279	15434709	Yellow	glycosyl hydrolase family 17 protein
		Gh_D06G0494	D06_7146007	intergenic	T	C	Ghir_D06G005570.1	D06	7896764	7899179	Turquoise	heptahelical transmembrane protein1
		Gh_D09G0438	D09_20646727	intergenic	T	G	Ghir_D09G004750.1	D09	21825189	21828396	Brown	subtilase 1.3
		Gh_D09G1018	D09_36121996	intergenic	G	A	Ghir_D09G011010.1	D09	37971256	37972729	Blue	FASCICLIN-like arabinogalactan-protein 12
		Gh_A08G0823	A08_41457601	intergenic	C	T	Ghir_A08G009510.1	A08	40570708	40573583	Blue	glucuronidase 3
			A08_41417038	intergenic	T	C						
			A08_41457669	intergenic	C	T						
		Gh_A12G1503	A12_74507587	nonsynonymous	T	A	Ghir_A12G017450.2	A12	93094640	93096349	Red	myb domain protein 16
	Lint index	Gh_D09G1281	D09_39936149	intergenic	T	C	Ghir_D09G013920.2	D09	41980796	41983294	Brown	Hypothetical protein, Interacts with GL2
		Gh_A08G0823	A08_41417038	intergenic	T	C	Ghir_A08G009510.1	A08	40570708	40573583	Blue	glucuronidase 3
		Gh_A12G1503	A12_74507587	nonsynonymous	T	A	Ghir_A12G017450.2	A12	93094640	93096349	Red	myb domain protein 16
	Fiber Weight Per Ball	Gh_D09G0438	D09_20662800	intergenic	A	G	Ghir_D09G004750.1	D09	21825189	21828396	Brown	subtilase 1.3

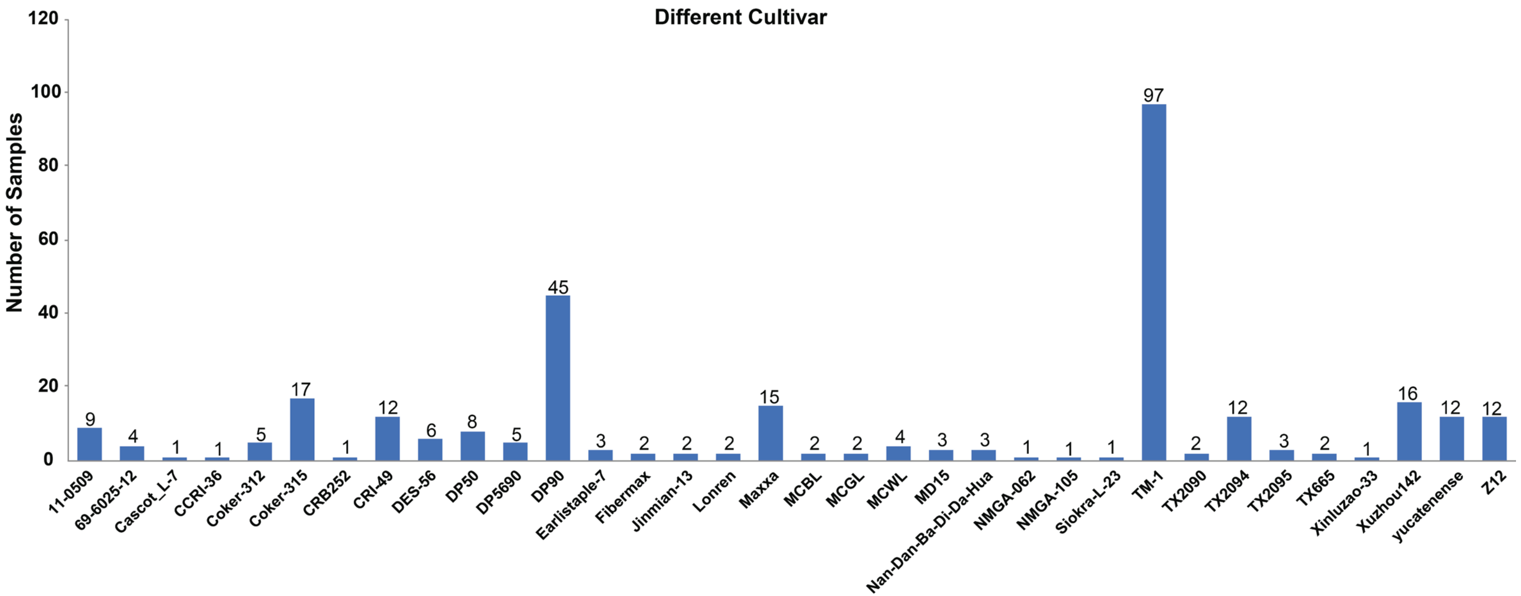

Supplementary Figure 1 Different cotton cultivars that were compiled during the initial screening processes

#GO Term	P-Value
protein storage vacuole	5.03E-05
storage vacuole	5.03E-05
seed maturation	0.001055782
nutrient reservoir activity	0.001193058
multicellular organismal reproductive process	0.004395418
plant-type vacuole	0.004528074
multicellular organism reproduction	0.004798919
mitochondrial electron transport, NADH to ubiquinone	0.007615557
developmental maturation	0.008253343
NADH dehydrogenase (quinone) activity	0.012913548
NADH dehydrogenase (ubiquinone) activity	0.012913548
NADH dehydrogenase activity	0.017433233
mitochondrial ATP synthesis coupled electron transport	0.019685677
oxidative phosphorylation	0.020435399
ATP synthesis coupled electron transport	0.020435399
respiratory electron transport chain	0.021933204
oxidoreductase activity, acting on NAD(P)H, quinone	0.023428827
cobalt ion binding	0.030874331
response to acid chemical	0.042309401

#KEGG Term	P-Value
Oxidative phosphorylation	5.98E-09
Metabolic pathways	7.30E-05
Photosynthesis	0.057955366
Phenylpropanoid biosynthesis	0.066877459
Ribosome	0.147915692
Biosynthesis of secondary metabolites	0.381178994

Supplementary Figure 2 GO and KEGG pathway enrichment of assembled supertranscripts

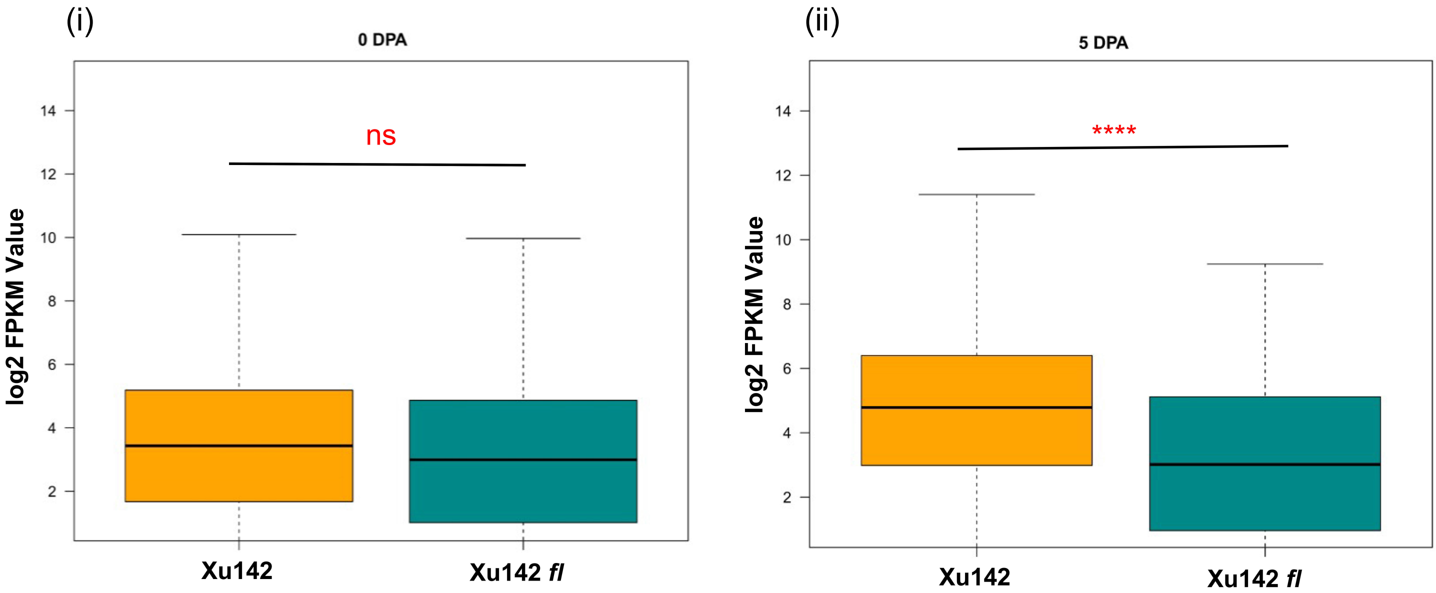

Supplementary Figure 3 Expression level quantification of upregulated differentially expressed genes in wild (Xu142) and fiberless mutant (Xu142 *fl*) cotton cultivar at (i) 0 DPA and (ii) 5 DPA fiber developmental stages

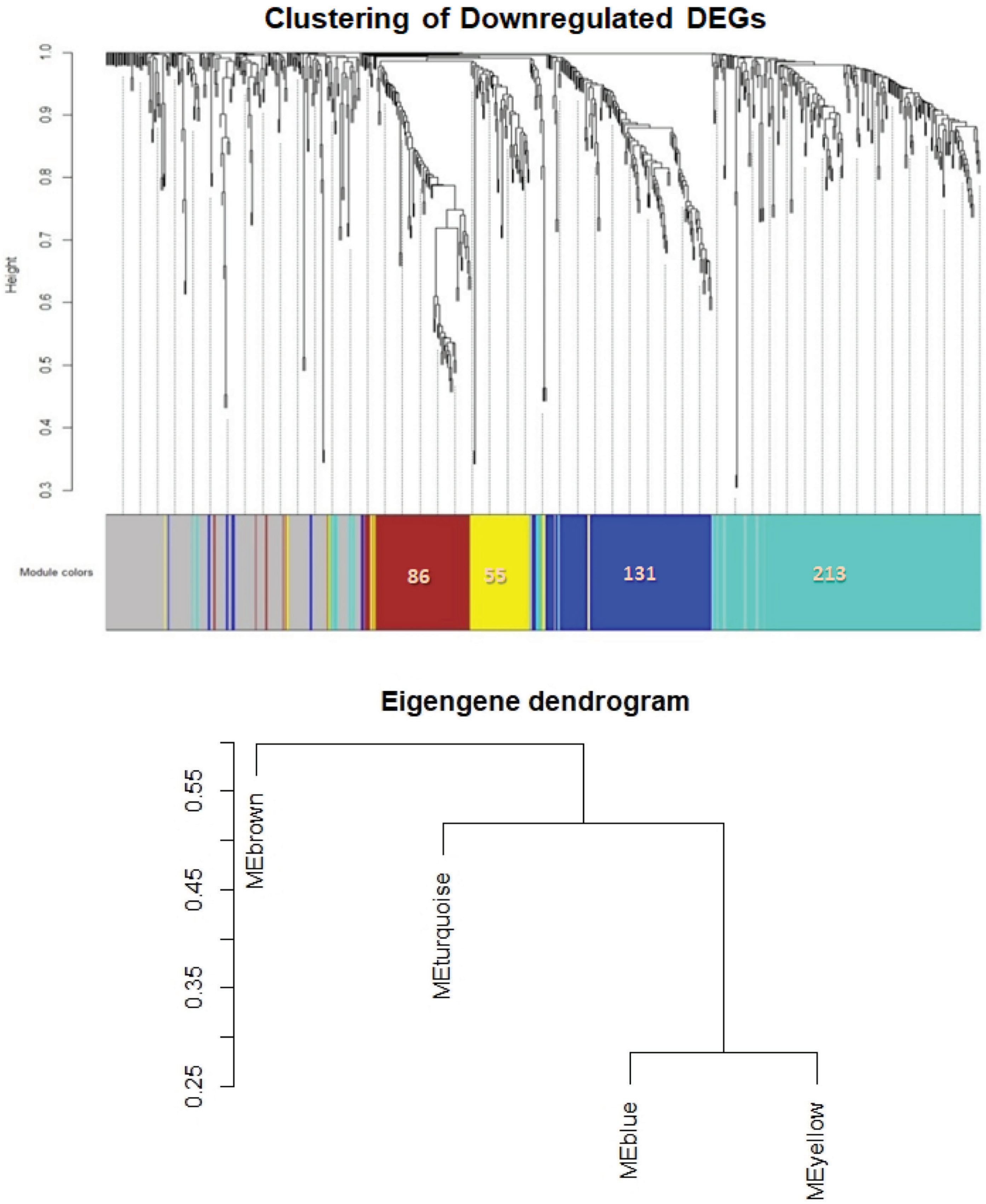

Supplementary Figure 4 Module wise clustering of down-regulated genes with their eigengene dendrogram

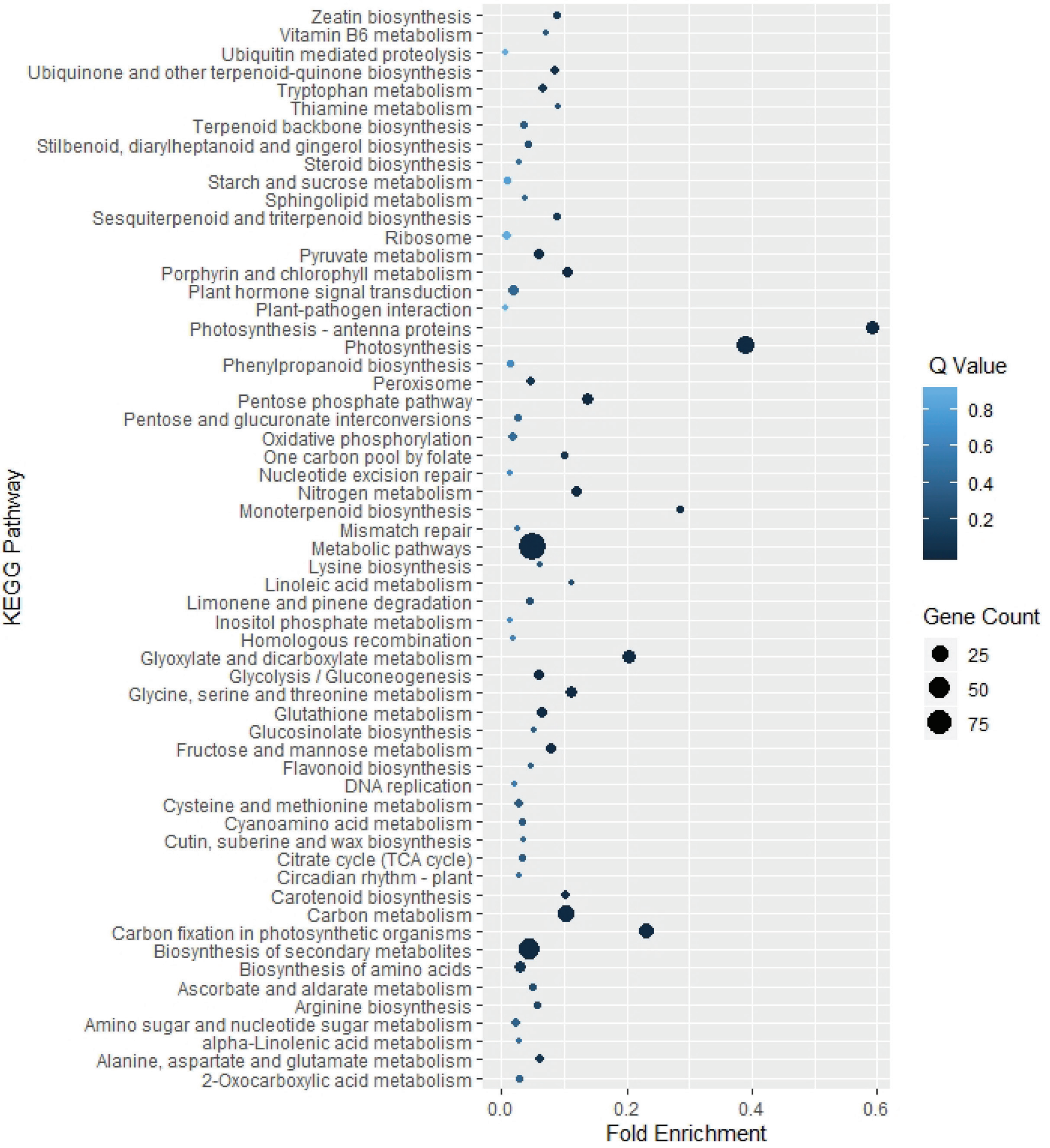

Supplementary Figure 5 KEGG pathway enrichment of the down regulated genes

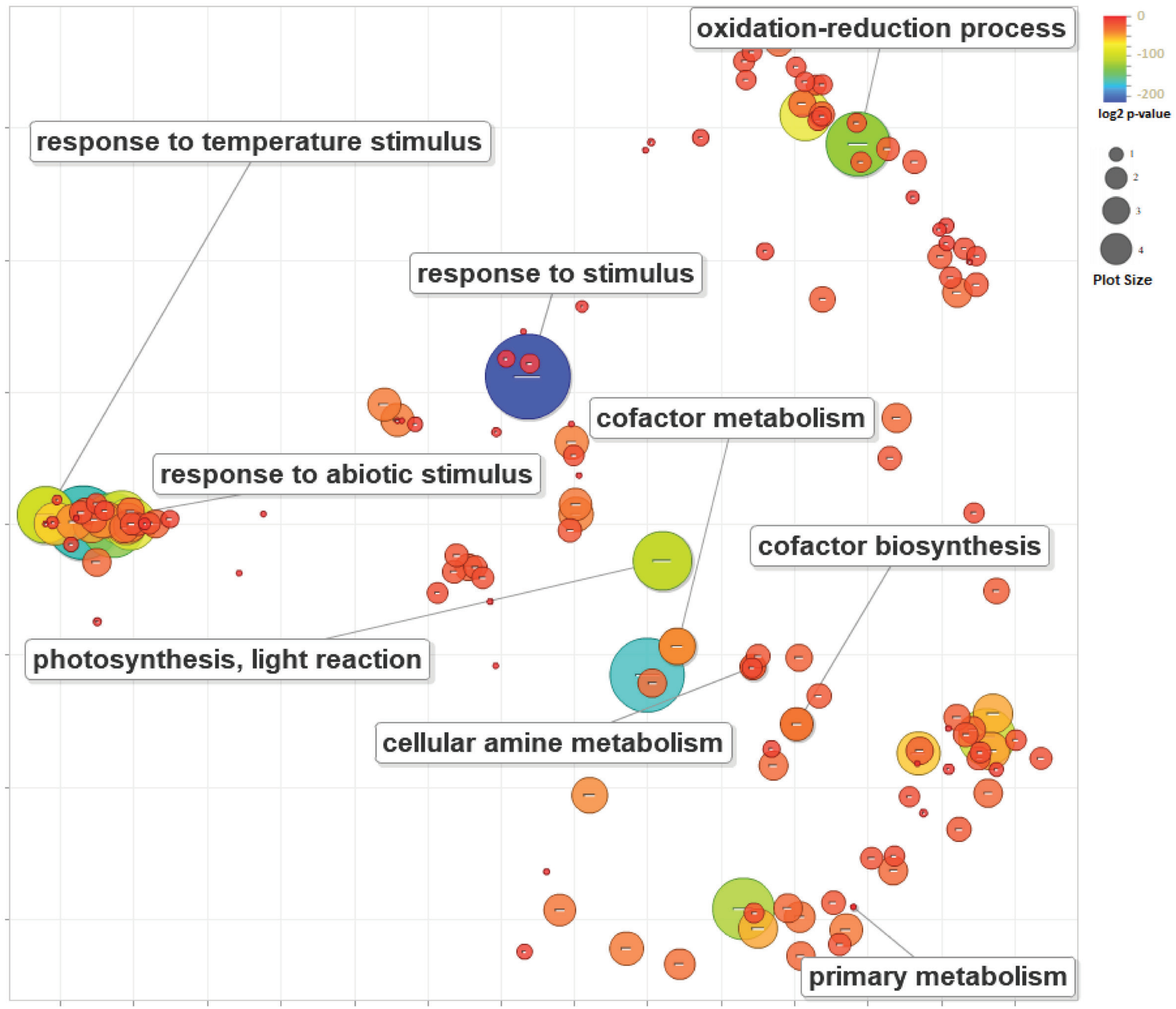

Supplementary Figure 6 Gene Ontology enrichement of the down regulated genes

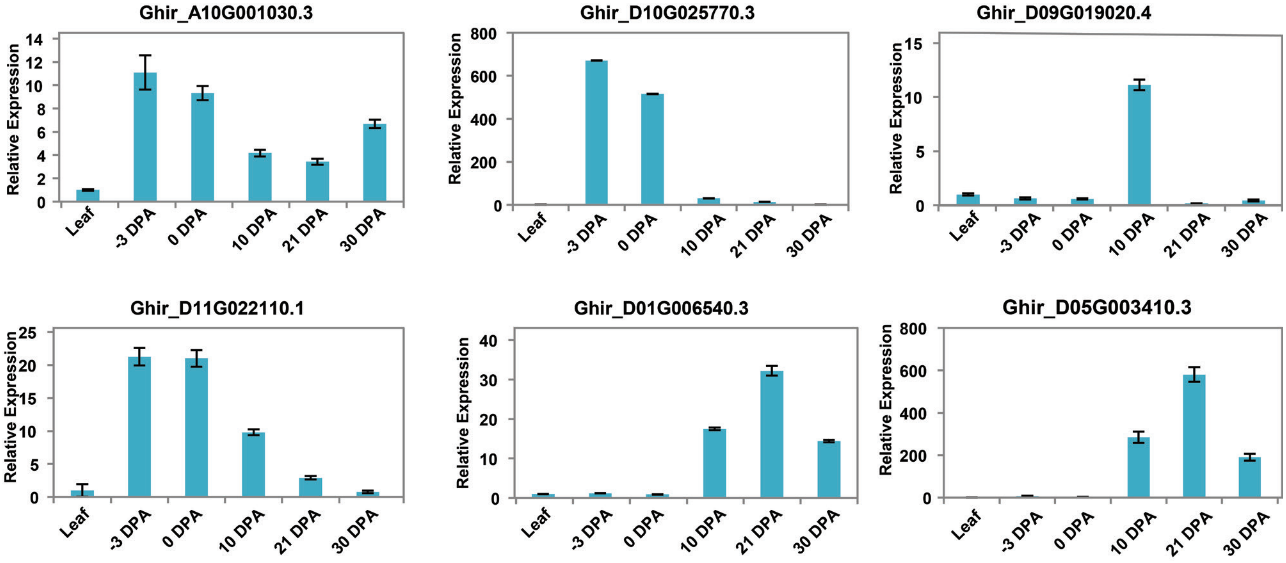

Supplementary Figure 7 Real-time expression validation of six selected Exclusive Expressed Transcripts (top five highly expressed transcripts and one low expressed transcript) in leaf and different fiber developmental stages viz, -3 DPA, 0 DPA, 10 DPA, 21 DPA and 30 DPA

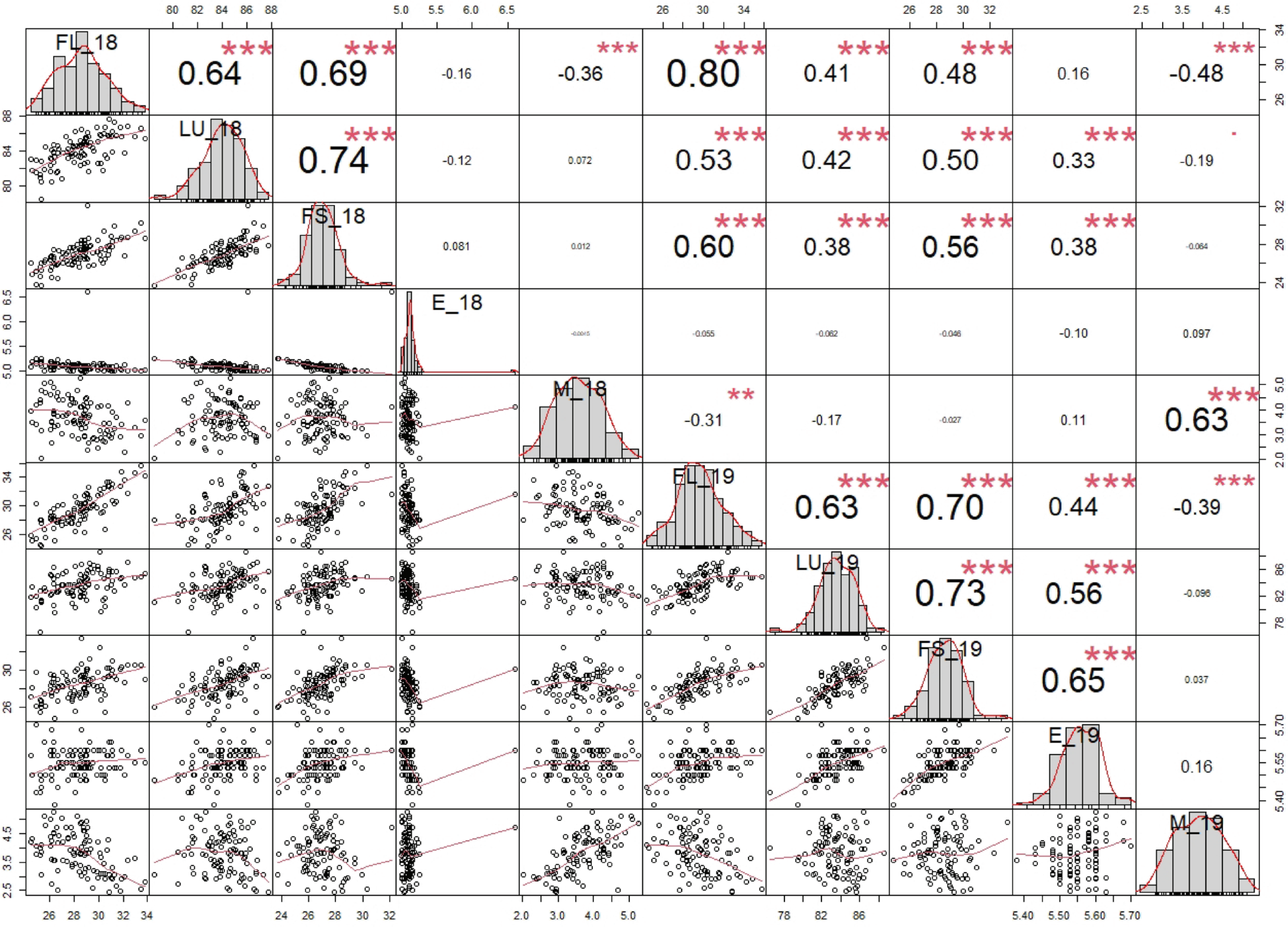

Supplementary Figure 8 Pearson Correlation analysis of 100 cotton genotypes in 2018 and 2019 year for different fiber-related traits

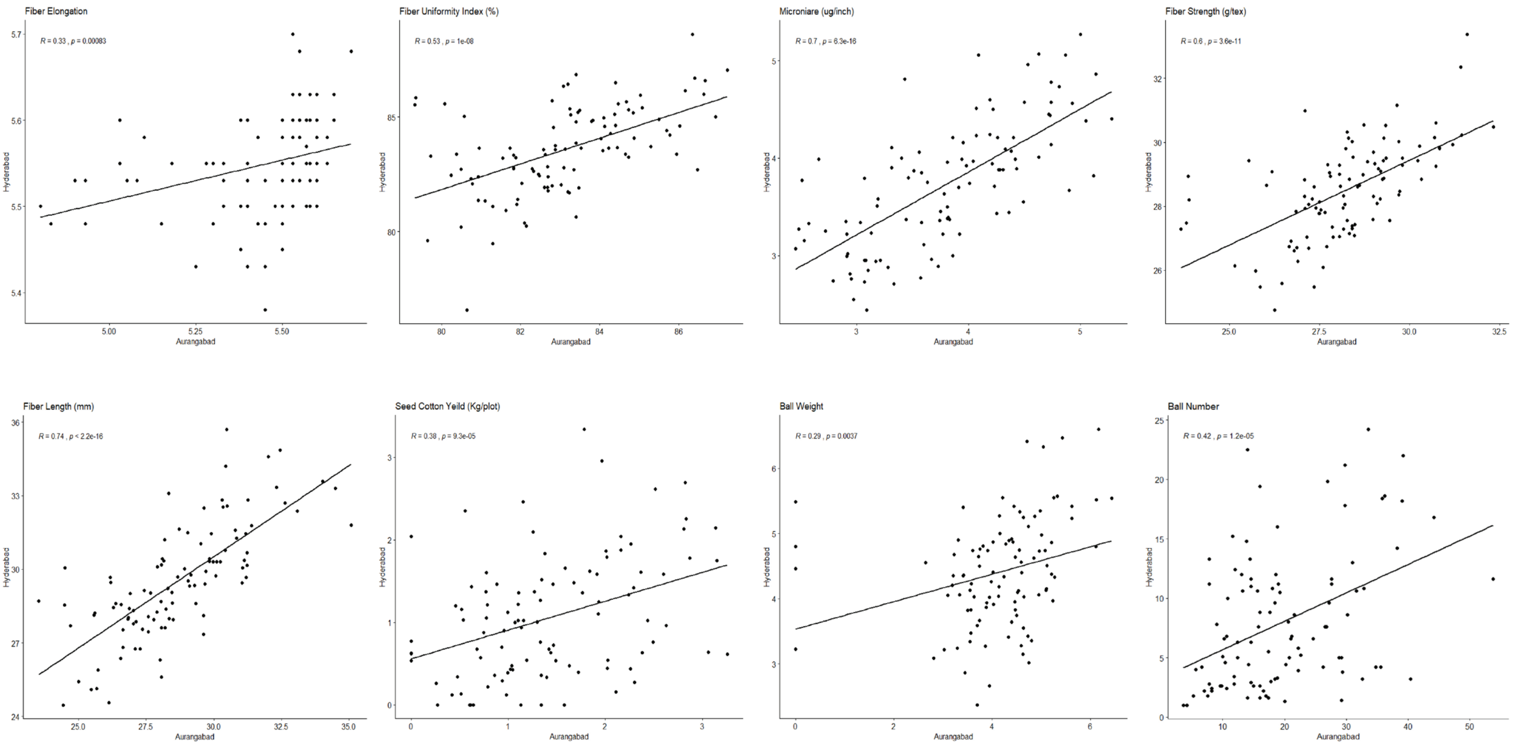

Supplementary Figure 9 Pearson Correlation analysis of 100 cotton genotypes at Hyderabad and Aurangabad city of India for different fiber-related traits
